# Cancer mortality and competing causes of death in older adults with cancer: A prospective, multicentre cohort study (ELCAPA‐19)

**DOI:** 10.1002/cam4.6639

**Published:** 2023-11-08

**Authors:** Déborah Assouan, Elena Paillaud, Philippe Caillet, Amaury Broussier, Emmanuelle Kempf, Maxime Frelaut, Etienne Brain, Emmanuelle Lorisson, Clelia Chambraud, Sylvie Bastuji‐Garin, Olivier Hanon, Florence Canouï‐Poitrine, Marie Laurent, Claudia Martinez‐Tapia

**Affiliations:** ^1^ Univ Paris Est Creteil, INSERM, IMRB Creteil France; ^2^ Department of Hematology Amiens University Hospital Amiens France; ^3^ Department of Geriatrics APHP (Assistance Publique–Hôpitaux de Paris), Georges Pompidou European Hospital Paris France; ^4^ Department of Geriatrics APHP, Henri Mondor/Emile Roux Hospitals Limeil‐Brevannes France; ^5^ Department of Medical Oncology APHP, Henri‐Mondor Hospital Creteil France; ^6^ Department of Medical Oncology Gustave Roussy Villejuif France; ^7^ Department of Medical Oncology Institut Curie Saint‐Cloud France; ^8^ Department of Geriatrics CHIC Creteil France; ^9^ Clinical Research Unit APHP, Henri‐Mondor Hospital Creteil France; ^10^ Public Health Department APHP, Henri‐Mondor Hospital Creteil France; ^11^ APHP, Broca Hospital Paris France

**Keywords:** cancer, cause of death, geriatric assessment

## Abstract

**Background:**

In older patients with cancer, comorbidities compete with cancer for cause of death. The objectives were to evaluate cancer mortality and factors associated, according to metastatic status.

**Methods:**

Between 2007 and 2014, patients with cancer aged ≥70 referred for pre‐therapeutic geriatric assessment (GA) were included through the ELCAPA prospective cohort study. The underlying cause of death was defined according to the International Classification of Diseases, 10th Revision. The World Health Organisation definition was used to categorise the cause of death as cancer versus another disease (e.g. cardiovascular disease, infectious disease, etc.) Competing risk models were used.

**Results:**

Mean (SD) age of the 1445 included patients was 80.2 (5.8) and 48% were women. Most common tumour sites were colorectal (19%), breast (17%) and urinary (15%); 773 patients (49%) had metastases. After a 34‐month median follow‐up, 706 cancer deaths were observed among 843 deaths. The 6‐month and 3‐year cancer mortality rates (95% CI) were 12% (9–15) and 34% (29–38) for non‐metastatic patients and 43% (39–47) and 79% (75–82) for metastatic patients, respectively. Dependency in activities of daily living and comorbidities were associated with 6‐month and 3‐year cancer mortality in non‐metastatic (adjusted subhazard ratio [aSHR] = 1.68 [0.99–2.85] and 1.69 [1.16–2.45]; and 1.98 [1.08–3.63] and 3.38 [1.47–7.76], respectively) and metastatic patients (aSHR = 2.81 [2.01–3.93] and 2.95 [2.14–4.07]; and 1.63 [1.18–2.25] and 2.06 [1.39–3.05], respectively). Impaired Timed‐Get‐Up‐and‐Go test was associated with 6‐month and 3‐year cancer mortality in metastatic patients (aSHR = 1.5 [1.06–2.12] and 1.38 [1.06–1.81], respectively). Obesity was negatively associated with 3‐year cancer death in non‐metastatic (aSHR = 0.53 [0.29–0.97]) and metastatic patients (aSHR = 0.71 [0.51–1.00]).

**Conclusions:**

The majority of older adults with cancer referred for pre‐therapeutic GA die from cancer. Geriatric parameters are independently associated with cancer mortality and should be considered for prognosis assessment, decision‐making and care.

## Graphical abstract

The majority of older adults with cancer referred for pre‐therapeutic GA died from cancer and not from other causes. An altered general status, loss of independency and co‐morbidities were independently associated with cancer death at 6 months and at 3 years, regardless of metastatic status; mobility impairment was independently associated with cancer death at 6 months and at 3 years in metastatic patients.

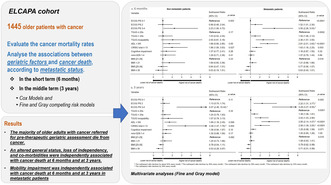

## 1 INTRODUCTION

Cancer is a common disease in the older adult, 60% of all cases occurring after the age of 65^1^ and 30% after the age of 75.^2^ The decision‐making process in cancer treatment is based on the selection of the best treatment option, patient information and physician‐patient dialogue. It requires an assessment of the individual's prognosis by making a distinction between cancer mortality and other causes of deaths, especially in older patients for whom risk of death related through comorbid conditions is higher than in middle‐aged adults.^3^, ^4^, ^5^

Numerous studies analysed the predictive value of oncological, clinical and biological factors for all‐cause mortality in older patients with cancer.^6^, ^7^, ^8^ Conversely, few assessed cancer‐specific mortality, and those which did, focused on a limited number of tumour types and clinical factors.^9^, ^10^ In clinical setting, one study demonstrated that age and comorbidities were associated with breast cancer‐specific mortality.^9^ In community‐dwelling setting, a recent study demonstrated that frailty phenotype predicted independently cancer mortality after adjustment for socio‐demographics and health‐related variables in US older people.^11^ However, to our best of knowledge, no study was conducted in clinical setting with a variety of tumour types, and the prognosis value of specific geriatric factors regarding cancer mortality in older patients with cancer is unknown. Moreover, evaluating specific factors associated with cancer mortality in metastatic and non‐metastatic patients is necessary, given the differences in the clinical profiles, mortality risk, approach to treatment and the implications for cancer specific mortality.^12^, ^13^ Also, the adverse effect of metastatic status differs significantly across tumour sites.^14^

The objectives were therefore to evaluate the cancer mortality rate and to analyse the associations between geriatric factors and cancer death, according to metastatic status.

## 2 PATIENTS AND METHODS

### 2.1 Patients and design

The ELCAPA non‐interventional, observational and prospective cohort study includes patients aged ≥70 years with a solid tumour or haematological malignancy, referred by the oncologist, or specialist for multidimensional geriatric assessment (GA) at one of the 19 geriatric oncology clinics in the Paris area (France). The ELCAPA cohort was created using an age inclusion criterion of ≥70 years, as this is the cut‐off recommended by national and international societies to perform a GA in this population.^15^, ^16^, ^17^ The present ELCAPA‐19 study included consecutive ELCAPA patients enrolled between January 2007 and December 2014 in eight investigation centres for whom data on metastatic status and follow‐up was available. All patients received oral presentation of the study by the geriatrician and a patient information leaflet. All patients provided oral informed consent prior to inclusion. The study was approved by the local institutional review board (CPP Ile‐de‐France I, Paris, France). The ELCAPA cohort study is registered in the ClinicalTrials.gov database (NCT02884375). The study report complies with the STROBE guidelines for observational studies.

### 2.2 Data collection

Data collected in the ELCAPA cohort include age, sex, Eastern Cooperative Oncology Group (ECOG) performance status (PS), cancer‐related characteristics (tumour site; metastatic status: no metastases and with metastases or haematological malignancies; cancer treatment: curative or palliative treatment or exclusive supportive care; previous history of cancer), and geriatric data (collected from the GA). The GA, performed by a geriatrician at baseline covers six domains (dependency, mobility, cognition, mood, nutritional status and comorbidity), based on validated tests and scores.^18^ Dependency is defined by an activities of daily living (ADL) score ≤5/6. Mobility impairment is defined as a Timed‐Get‐Up‐and‐Go (TGUG) completion time >20 s or inability to perform the test. Cognitive impairment is defined by a Mini‐Mental State Examination (MMSE) score <24/30 or a history of dementia. A mini‐Geriatric Depression Scale (mini‐GDS) score ≥1/4 is suggestive of a depressive mood disorder. The patient's body mass index (BMI) is classified as follows: <21 kg/m^2^ = underweight, between 21 kg/m^2^ and 24.99 kg/m^2^ = normal weight, between 25 kg/m^2^ and 29.99 kg/m^2^ = overweight, and ≥30 kg/m^2^ = obesity. Comorbidity burden is assessed by the Cumulative Illness Rating Scale for Geriatrics (CIRS‐G) and was considered high as a total CIRS‐G score above the median value in the study population (≥13).

### 2.3 Cancer mortality

The main endpoint was cancer mortality in the short term (6 months) and middle term (3 years). The underlying cause of death (UCD) is defined by the World Health Organisation: ‘the disease or injury that initiated the train of morbid events leading directly to a person's death or the circumstances of the accident or violence which produced the fatal injury’. UCD information was obtained from the French Epidemiological Centre for the Medical Causes of Death (CépiDC), a nationwide database. For each death occurring on French territory, a physician completes an international death certificate form; from this certificate, the CépiDC assigns a single UCD to each death according to internationally adopted rules, coded according to the International Classification of Diseases, 10th Revision. This UCD selection and coding process was performed by coding software's and experienced coders. To identify the UCD of each patient, we linked the ELCAPA cohort to the CépiDC.^19^ In the present study, we used the WHO definition to categorise the cause of death as either cancer or another disease (e.g. cardiovascular disease, infectious disease, etc.).

### 2.4 Statistical analysis

Baseline demographic, cancer and geriatric characteristics, and cause of death, were described as number and percentage for qualitative variables, and the mean (standard deviation; SD) or median (interquartile range), where appropriate, for quantitative variables, according to metastatic status. A Fine and Gray competing risk model^20^ with cancer death as the event of interest and non‐cancer death as a competing event was used to estimate the cancer mortality rates at 6 months and 3 years after enrolment with 95% confidence intervals (CIs) and the cumulative incidence function for cancer deaths. We also examined the effect of baseline demographic, oncological and geriatric characteristics on cancer deaths by calculating the subhazard ratio (SHR). All variables with a *p*‐value below 0.20 in the univariate analysis were considered in the multivariate analysis. Collinearity between PS and CIRS‐G, and between TGUG and ADL, was assessed. A manual stepwise analysis was done to identify confounders. Associations between the above factors and cancer deaths were also analysed using a Cox‐hazard proportional model and reported as cause‐specific hazard ratio (CSHR).^21^ The proportional hazards assumption of the Cox regression was tested. Interactions between time and tested factors were assessed. Assuming data were missing at random, variables with missing values were imputed using a multivariate chain equation imputation procedure.

#### 2.4.1 Sensitivity analyses

In order to test the stability of our results, we performed Fine and Gray competing risk models in patients with a solid tumour and specific treatment for cancer.

All tests were two‐sided and the statistical significance threshold was set at *p* < 0.05. The data were analysed using Stata software (release 13.0, StataCorp LLC, College Station, TX).

## 3 RESULTS

### 3.1 Patient characteristics

Between January 2007 and December 2014, of the 1678 patients recruited in the ELCAPA cohort, 1445 patients had complete metastatic status data and were included in the present analysis. Mean (SD) age was 80.2 ± 5.8 years and 695 (48%) were women. The most common primary tumours were colorectal (19%), breast (17%) and urinary tract (15%). Forty‐nine percent of patients with solid tumours had metastases. The main characteristics of the population are summarised in Table 1. Patients with metastases were more likely to have a poor PS, dependencies for ADLs, reduced mobility, cognitive impairment, risk for depression and high burden of comorbidities, compared to patients with no metastases.

**TABLE 1:** Demographic, oncologic and geriatric characteristics of the study population at inclusion, overall and by metastatic status.

Characteristic	Number of patients evaluated	*N* = 1445	Metastases		*p*‐value
			No (*n* = 672)	Yes^a^ (*n* = 773)	
Age, years	1445				
Mean (SD)	–	80.2 ± 5.8	80.2 (±5.9)	80.2 (±5.7)	0.99
Classes, *n* (%)	–				
<80	–	660 (45.7)	310 (46.1)	350 (45.3)	0.74
80–89	–	688 (47.6)	314 (46.7)	374 (48.4)	
≥90	–	97 (6.7)	48 (7.1)	49 (6.3)	
Sex, female, *n* (%)	1445	695 (48.1)	342 (50.9)	353 (45.7)	**0.047**
Tumour site, *n* (%)	1445				
Colorectal	–	277 (19.2)	139 (20.7)	138 (17.9)	**<0.0001**
Breast	–	245 (17.0)	156 (23.2)	89 (11.5)	
Urinary tract	–	211 (14.6)	118 (17.6)	93 (12.0)	
UDT and liver	–	154 (10.7)	84 (12.5)	70 (9.0)	
Prostate	–	167 (11.6)	87 (13.0)	80 (10.4)	
Haematological	–	121 (8.4)	0 (0.0)	121 (15.7)	
Pancreas	–	80 (5.5)	33 (4.9)	47 (6.1)	
Lung	–	43 (3.0)	13 (1.93)	30 (3.9)	
Skin	–	37 (2.6)	9 (1.3)	28 (3.6)	
Unknown primary cancer	–	44 (3.0)	2 (0.3)	42 (5.4)	
Others^b^	–	66 (4.6)	31 (4.6)	35 (4.5)	
History of cancer, *n* (%)	1439	336 (23.4)	138 (20.6)	198 (25.7)	**0.023**
Cancer treatment decision, *n* (%)	1236				
Curative	–	561 (45.4)	410 (72.4)	151 (22.5)	**<0.0001**
Palliative care	–	383 (31.0)	63 (11.1)	320 (47.8)	
Supportive care alone	–	292 (23.6)	93 (16.4)	199 (29.7)	
ECOG‐PS, *n* (%)	1436				
0–1	–	699 (48.7)	411 (61.6)	288 (37.5)	**<0.0001**
2	–	265 (18.5)	115 (17.2)	150 (19.5)	
3–4	–	472 (32.8)	141 (21.1)	331 (43.0)	
Dependency for ADLs (≤ 5 out of 6), *n* (%)	1425	476 (33.4)	164 (24.6)	312 (41.1)	**<0.0001**
Mobility, TGUG, *n* (%)	1406				
≤20 s	–	772 (54.9)	426 (65.3)	346 (45.9)	**<0.0001**
>20 s	–	448 (31.9)	176 (27.0)	272 (36.1)	
Unable	–	186 (13.2)	50 (7.7)	136 (18.0)	
Cognitive impairment, *n* (%)^c^	1357	371 (27.3)	152 (23.9)	219 (30.4)	**0.008**
Abnormal mini‐GDS score (≥1 out of 4), *n* (%)	1262	428 (33.9)	163 (28.0)	265 (39.0)	**<0.0001**
BMI (kg/m^2^), *n* (%)	1385				
<21	–	491 (35.5)	223 (34.2)	268 (36.6)	**<0.0001**
(21–25)	–	233 (16.8)	71 (10.9)	162 (22.1)	
(25–30)	–	482 (34.8)	257 (39.4)	225 (30.7)	
≥30	–	179 (12.9)	101 (15.5)	78 (10.6)	
CIRS‐G total score	1328				
Median (IQR)	–	13 (9–17)	12 (8–15)	14 (10–19)	**<0.0001**
≥13, *n* (%)	–	674 (50.8)	248 (40.9)	423 (59.2)	**<0.0001**

Abbreviations: ADL, activities of daily living; BMI, body mass index; CIRS‐G, Cumulative Illness Rating Scale for geriatrics; ECOG‐PS, Eastern Cooperative Group performance status; GDS: Geriatric Depression Scale; IQR, interquartile range; SD, standard deviation; TGUG, Timed‐Get‐Up‐and‐Go Score; UDT, upper digestive tract.

Bold values denote statistical significance at the *p* <0.05 level.

^a^ Solid and haematological cancers.

^b^ Metastases no/yes: gynaecological (*n* = 8/16), sarcoma (*n* = 4/12), brain (*n* = 8/0), head and neck (*n* = 6/3), thyroid (*n* = 1/2), others (*n* = 4/2).

^c^ Cognitive impairment was defined as a Mini‐Mental State Examination score below 24 out of 30 or a history of cognitive disorders.

### 3.2 Incidences of cancer deaths and non‐cancer deaths

With a median follow‐up of 34 months, 843 deaths occurred, 706 (84%) of which were due to cancer, 503/561 (90%) in patients with metastasis and 203/282 (72%) in patients without.

The cancer mortality rates at 6 months and 3 years were 12% (95% CI, 9%–15%) and 34% (95% CI, 29%–38%) for non‐metastatic cancers, and 43% (95% CI, 39%–47%) and 79% (95% CI, 75%–82%) for metastatic cancers, respectively. The cumulative incidence curves of cancer deaths are shown in Figure S1. Cancer mortality rates by tumour site and metastatic status are shown in Table 2.

**TABLE 2:** Cancer mortality rates (95% CI) by tumour site and metastatic status.

	Cancer mortality rates (95% CI)			
	Without metastases		With metastases	
Tumour site	6 months	3 years	6 months	3 years
Colorectal	8 (5–14)	25 (16–35)	31 (24–40)	78 (69–85)
Breast	1 (0–5)	6 (3–13)	26 (18–37)	73 (61–84)
Urinary	19 (13–27)	46 (34–57)	42 (32–53)	81 (71–90)
UDT and liver	20 (13–31)	65 (52–78)	61 (50–73)	92 (83–97)
Prostate	2 (1–9)	13 (7–24)	36 (26–48)	90 (70–89)
Pancreas^a^	27 (14–47)	75 (56–91)	75 (61–87)	–
Lung	16 (4–51)	55 (23–91)	90 (73–98)	95 (79–100)
Skin^b^	–	–	67 (49–84)	79 (62–92)
Unknown primary^c^	NA	NA	54 (39–70)	90 (76–97)
Haematological cancer	NA	NA	32 (25–42)	53 (43–63)
Other	35 (20–57)	63 (40–85)	39 (24–58)	66 (47–84)

*Note*: Results are given as mortality rates (95% CI).

Abbreviations: NA, not applicable; UDT, upper digestive tract.

^a^ At 3 years, there were no more at‐risk metastatic patients (all the patients with metastatic pancreatic cancer had died or were lost to follow‐up).

^b^ At 6 months, there were no more at‐risk non‐metastatic patients (all of the nine patients with non‐metastatic skin cancer had died or were lost to follow‐up).

^c^ Since only two patients had a non‐metastatic unknown primary cancer, reliable estimation of the cancer mortality rate was not possible.

### 3.3 Univariate and multivariate analyses of factors associated with cancer death, by metastatic status

At 6 months and at 3 years, palliative care, supportive care alone, PS 3–4, dependency for ADLs, abnormal TGUG time or inability to perform TGUG, impaired cognitive status, abnormal mini‐GDS score, and comorbidities (CIRS‐G ≥ 13) were significantly associated with cancer death (*p* < 0.05 for all) regardless of metastatic status (Table 3). Male sex was significantly associated with cancer death only at 3 years. Obesity was associated with a lower number of cancer deaths at 6 months and 3 years, regardless of metastatic status (*p* < 0.05) (Table 3). Overweight was associated with a lower number of cancer deaths in patients with metastasis, at 6 months and 3 years, and in patients without metastasis, only at 3 years. Older age was significantly associated with cancer death at 3 years in non‐metastatic patients. History of previous cancer was significantly associated with cancer death only in metastatic patients, at 6 months and was not significant (*p* = 0.093) at 3 years. These variables were included in the multivariate analyses.

**TABLE 3:** Univariate analyses of factors associated with cancer death (Fine‐Gray model), by metastatic status.

	At 6 months						At 3 years					
	Non‐metastatic			Metastatic			Non‐metastatic			Metastatic		
	(74 cancer deaths)			(306 cancer deaths)			(173 cancer deaths)			(491 cancer deaths)		
Variables	SHR	95% CI	*p*‐value	SHR	95% CI	*p*‐value	SHR	95% CI	*p*‐value	SHR	95% CI	*p*‐value
Age, years												
<80	1 (reference)		0.12	1 (reference)		0.24	1 (reference)		0.006	1 (reference)		0.43
80–89	1.33	0.82–2.17		1	0.8–1.27		1.63	1.19–2.25		1.05	0.88–1.26	
≥90	2.28	1.03–5.05		1.43	0.93–2.21		1.89	1.02–3.5		1.3	0.87–1.95	
Sex, female	0.84	0.53–1.32	0.45	0.8	0.63–1	0.05	0.67	0.49–0.9	0.008	0.79	0.66–0.95	0.011
Tumour site												
Colorectal	1 (reference)		<0.001	1 (reference)		<0.001	1 (reference)		<0.001	1 (reference)		<0.001
Breast	0.08	0.01–0.63		0.81	0.48–1.37		0.24	0.1–0.55		0.82	0.6–1.14	
Urinary tract	2.3	1.11–4.78		1.47	0.94–2.29		2.88	1.79–4.62		1.3	0.96–1.77	
UDT and liver	2.41	1.1–5.27		2.66	1.73–4.1		3.15	1.93–5.15		2.3	1.68–3.17	
Prostate	0.27	0.06–1.23		1.25	0.76–2.05		0.5	0.24–1.07		1.1	0.78–1.55	
Pancreas	3.62	1.44–9.09		3.41	2.16–5.39		5.21	2.85–9.53		2.68	1.85–3.89	
Haematological	NA	NA		1.04	0.67–1.62		NA	NA		0.62	0.44–0.87	
Others^a^	4.61	2.12–9.99		2.37	1.62–3.47		4.27	2.42–7.52		1.68	1.26–2.26	
History of previous cancer	1.16	0.68–2.0	0.59	1.33	1.05–1.7	0.02	1.16	0.81–1.65	0.42	1.19	0.97–1.46	0.093
Cancer treatment decision												
Curative	1 (reference)		<0.001	1 (reference)		<0.001	1 (reference)		<0.001	1 (reference)		<0.001
Palliative care	5.87	2.93–11.7		2.53	1.6–4		4.43	2.9–6.77		1.96	1.52–2.52	
Supportive care alone	8.31	4.61–15.0		7.22	4.58–11.4		3.99	2.71–5.87		3.71	2.76–4.99	
ECOG–PS												
0–1	1 (reference)		<0.001	1 (reference)		<0.001	1 (reference)		<0.001	1 (reference)		<0.001
2	1.63	0.81–3.3		2.06	1.41–3.01		1.69	1.15–2.49		1.41	1.11–1.79	
3–4	5.67	3.39–9.47		5.52	4.1–7.45		3.03	2.13–4.32		2.78	2.27–3.41	
Dependency for ADLs (≤ 5 out of 6)	3.48	2.2–5.49	<0.001	3.45	2.74–4.35	<0.001	2.54	1.85–3.48	<0.001	2.12	1.76–2.55	<0.001
Mobility, TGUG												
≤20 s	1 (reference)		<0.001	1 (reference)		<0.001	1 (reference)		<0.001	1 (reference)		<0.001
>20 s	2.33	1.38–3.94		2.67	2.02–3.54		1.97	1.41–2.74		1.79	1.47–2.18	
Unable	7.3	3.99–13.3		6.42	4.74–8.72		3.72	2.22–6.24		3.68	2.79–4.85	
Cognitive impairment^b^	2.24	1.37–3.67	0.001	2.16	1.7–2.74	<0.001	1.81	1.29–2.53	0.001	1.77	1.44–2.17	<0.001
Abnormal mini‐GDS score (≥1 out of 4)	1.95	1.18–3.21	0.009	1.66	1.3–2.13	<0.001	1.76	1.26–2.46	0.001	1.28	1.05–1.56	0.016
BMI (kg/m2)												
<21	1.32	0.67–2.59		1.43	1.08–1.9		1.05	0.65–1.7		1.17	0.92–1.5	
(21–25)	1 (reference)		0.053	1 (reference)		<0.001	1 (reference)		0.003	1 (reference)		<0.001
(25–30)	0.7	0.41–1.21		0.62	0.46–0.85		0.7	0.5–0.99		0.67	0.53–0.83	
≥30	0.36	0.14–0.92		0.54	0.33–0.86		0.39	0.23–0.68		0.64	0.47–0.88	
CIRS–G ≥ 13	4.91	2.8–8.61	<0.001	3.13	2.36–4.14	<0.001	2.44	1.79–3.33	<0.001	1.75	1.46–2.11	<0.001

Abbreviations: ADL, activities of daily living; BMI, body mass index; CI, confidence interval; CIRS‐G, Cumulative Illness Rating Scale for Geriatrics; ECOG‐PS, Eastern Cooperative Group performance status; GDS, Geriatric Depression Scale; SHR, subhazard ratio; TGUG, Timed‐Get‐Up‐and‐Go score; UDT, upper digestive tract.

^a^ Metastases no/yes: gynaecological (*n* = 8/16), sarcoma (*n* = 4/12), brain (*n* = 8/0), head and neck (*n* = 6/3), thyroid (*n* = 1/2), others (*n* = 4/2).

^b^ Cognitive impairment is defined as a Mini‐Mental State Examination below 24 out of 30 or a history of cognitive disorders.

Considering the collinearities between PS and CIRS‐G and between TGUG and ADL, we built two multivariate models: the first with PS and TGUG (Model 1) and the second with ADL and CIRS‐G (Model 2). Fine and Gray competing risk models and Cox models were built with PS, ADL and CIRS‐G as time‐dependent covariates.

**FIGURE 1:**
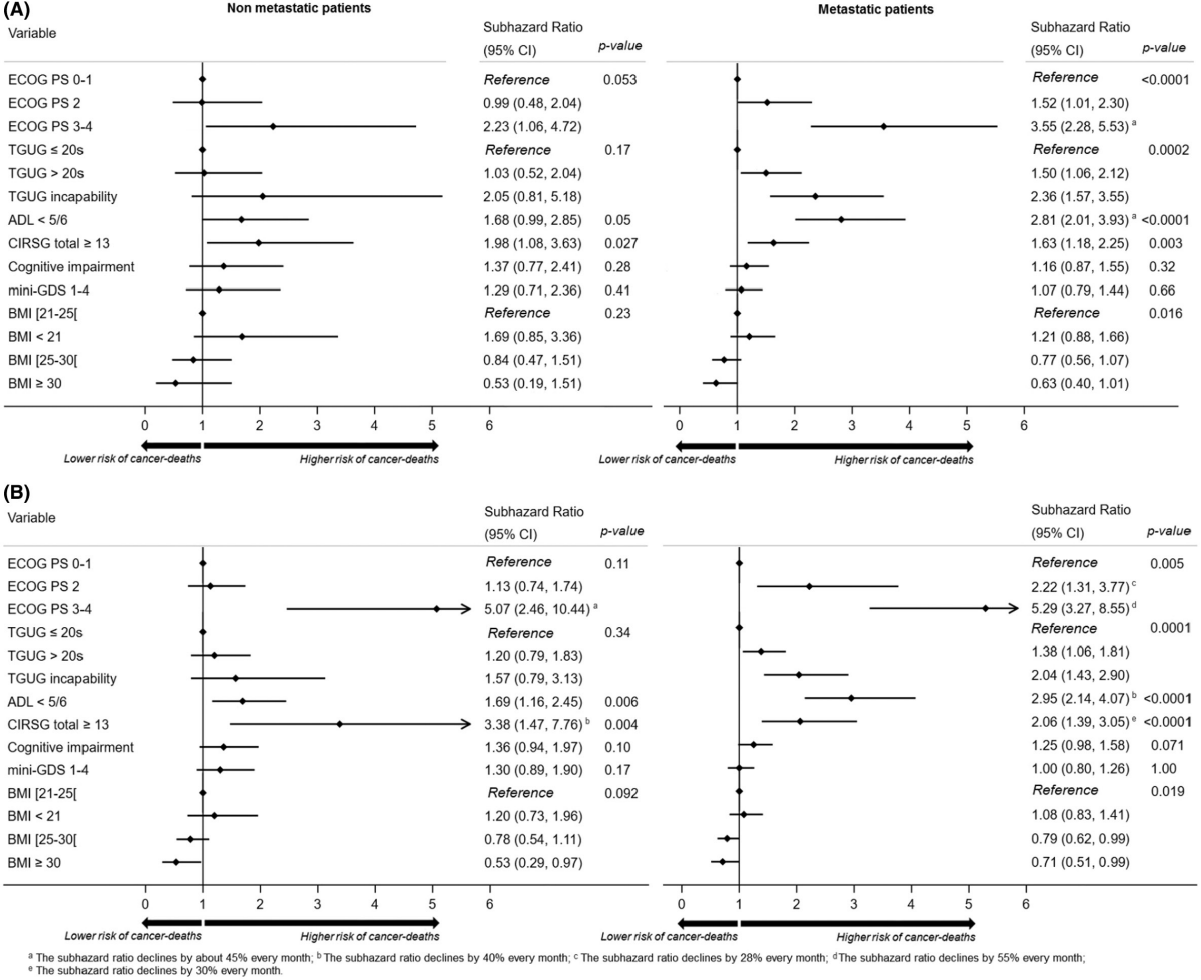
Geriatric factors associated with cancer death at (A) 6 months and (B) 3 years, in multivariate analyses (Fine and Gray model) by metastatic status. Adjusted subhazard ratios of cognitive impairment, mini‐GDS and BMI correspond to those from Model 1.

In non‐metastatic patients, an altered general status (PS 3–4), loss of independency (ADL score ≤5) and comorbidities (total CIRS‐G ≥ 13) were independently associated with cancer death at 6 months and 3 years, after adjusting for age, sex, tumour site, history of cancer and curative treatment vs. palliative care (Figure 1); obesity was independently associated with a lower risk of cancer death only at 3 years.

In metastatic patients, an altered general status (PS ≥2), an impaired mobility (TGUG >20 s or inability to perform TGUG), loss of independency (ADL score ≤5), and comorbidities (total CIRS‐G ≥ 13) were independently associated with cancer death at 6 months and 3 years; cognitive impairment was associated with cancer death at 3 years only in Model 2 (adjusted SHR, 1.35; 95% CI, 1.07–1.70; p‐value = 0.012). In contrast, overweight and obesity were independently associated with a lower risk of cancer death at 3 years. We found similar associations in a cause‐specific Cox proportional hazards model, with the exception of cognitive impairment, which in the Cox model was associated with a higher risk of cancer death at 3 years in metastatic patients, in the two models (Table 4). Significant interactions between time and comorbidities, ECOG‐PS and ADL were found and were taken into account in each model. The risk of cancer death related to the latter factors decreased with time. Similar results were found in complete‐case analysis (Fine‐Gray competing risk model and cause‐specific Cox proportional hazards model) at 6 months and 3 years (data not shown).

**TABLE 4:** Multivariate analyses of factors associated with cancer death: a cause‐specific Cox model.

	At 6 months						At 3 years					
	Non‐metastatic			Metastatic			Non‐metastatic			Metastatic		
Variable	aCSHR^a^	95%CI	*p*‐value	aCSHR^a^	95%CI	*p*‐value	aCSHR^a^	95%CI	*p*‐value	aCSHR^a^	95%CI	*p*‐value
ECOG‐PS												
0–1	1 (ref.)		0.076	1 (ref.)		<0.001	1 (ref.)		0.0003	1 (ref.)		<0.001
2	0.95	0.44–2.05		1.49	0.98–2.28		1.11	0.72–1.71		1.96^e^	1.16–3.3	
3–4	2.15	0.99–4.69		3.56^d^	2.24–5.67		4.54^d^	2.15–9.59		4.49^f^	2.79–7.22	
Mobility, TGUG												
≤20 s	1 (ref.)		0.17	1 (ref.)		0.0002	1 (ref.)		0.46	1 (ref.)		0.0001
>20 s	1.05	0.52–2.1		1.53	1.07–2.19		1.17	0.77–1.79		1.46	1.13–1.9	
Unable to perform the test	2.06	0.82–5.21		2.38	1.56–3.64		1.55	0.78–3.07		2.18	1.55–3.06	
Dependency for ADLs (≤5/6)^b^	1.71	0.98–2.99	0.059	2.85^d^	2.04–3.98	<0.001	1.67	1.15–2.43	0.007	2.84^h^	2.06–3.9	<0.001
CIRS‐G ≥13^b^	2.01	1.1–3.67	0.024	1.65	1.19–2.3	0.003	3.10^g^	1.41–6.8	0.005	1.98^e^	1.35–2.9	0.001
Cognitive impairment^c^	1.39	0.78–2.45	0.26	1.15	0.85–1.54	0.357	1.30	0.9–1.89	0.161	1.29	1.01–1.64	0.038
Abnormal mini‐GDS (≥1/4)	1.29	0.72–2.31	0.389	1.13	0.84–1.51	0.42	1.31	0.91–1.9	0.144	1.10	0.88–1.37	0.391
BMI (kg/m^2^)												
<21	1.76	0.86–3.62		1.24	0.92–1.68		1.23	0.74–2.04		1.11	0.86–1.42	
(21–25)	1 (ref.)		0.12	1 (ref.)		0.011	1 (ref.)		0.055	1 (ref.)		0.013
(25–30)	0.86	0.48–1.54		0.78	0.56–1.08		0.80	0.55–1.15		0.78	0.61–0.99	
≥30	0.52	0.19–1.4		0.64	0.39–1.03		0.51	0.29–0.92		0.71	0.5–0.99	

Abbreviations: aCSHR, adjusted Cox specific hazard model; ADL, activities of daily living; BMI, body mass index; CI, confidence interval; ECOG‐PS, Eastern Cooperative Group performance status; GDS, geriatric depressions; TGUG, Timed‐Get‐Up‐and‐Go score.

^a^ The Cox cause‐specific hazard model was adjusted for age, sex, tumour site, history of cancer, cancer treatment and all variables listed in the table except CIRS‐G and ADL (Model 1). Missing data were imputed using multivariate imputation by chained procedure.

^b^ CSHR of CIRS‐G and ADL were estimated in a Model 2 adjusted for age, sex, tumour site, history of cancer, cancer treatment and all variables listed in the table except ECOG‐PS and TGUG. Missing data were imputed using multivariate imputation by chained procedure.

^c^ Cognitive impairment is defined as a Mini‐Mental State Examination below 24 out of 30 or a history of cognitive disorders.

^d^ The CSHR declines by 40% every month.

^e^ The CSHR declines by 24% every month.

^f^ The CSHR declines by 45% every month.

^g^ The CSHR declines by 37% every month.

^h^ The CSHR declines by 31% every month.

### 3.4 Sensitivity analyses

Sensitivity analyses of patients with a solid tumour and specific treatment for cancer (*n* = 871) gave much the same results as the main analyses (Table S1). However, in non‐metastatic patients, an altered general status (PS 3–4) was not associated with cancer death (at 6 months and 3 years). Comorbidities (total CIRS‐G ≥ 13) and obesity were not associated either, at 3 years. In metastatic patients, obesity was not associated with a lower risk of cancer death at 3 years.

## 4 DISCUSSION

In older adults with cancer referred for pre‐therapeutic GA, the majority of deaths were attributed to cancer (84%). At 6 months, the cancer mortality rate was 12% (from 1% for breast cancer to 27% for pancreatic cancer) for non‐metastatic solid tumours, and 45% (from 26% for breast cancer to 90% for lung cancer) for metastatic solid tumours. The 3‐year cancer mortality rate was 34% (from 6% for breast cancer and 75% for pancreatic cancer) in non‐metastatic tumours, and 83% (from 73% for breast cancer to 95% for lung cancer) in metastatic tumours. In multivariate analysis adjusted for age, sex, tumour site/metastatic status, history of cancer and curative treatment vs. palliative care, an altered general status (PS 3–4), loss of independency (ADL score ≤5), and comorbidities (total CIRS‐G ≥ 13) were independently associated with cancer death in the short to middle term, regardless of metastatic status. Impaired mobility (TGUG >20 s or inability to perform TGUG) and an altered general status (PS = 2) were independently associated with cancer death in the short to middle term only in metastatic patients. Cognitive impairment was associated with cancer death (in one model) in the middle term only in metastatic patients. Overweight and obese patients were less likely to die from cancer than normal weight patients also in the middle term in metastatic patients, and obesity was also associated with a lower risk of cancer death in the middle term in non‐metastatic patients.

Few studies on causes of death are available and were performed mostly in prostate and breast cancer. Older men with non‐metastatic prostate cancer had a low 3‐year (5% to 20%) or 5‐year cancer mortality rate (8%), which corresponds to our value of 13%.^10^, ^22^ In breast cancer, Schonberg et al showed a 5‐year cancer mortality rates of 11–18% for stage I, 27–47% for stage II and 63–76% for stage III/IV tumours.^23^ Even with different stages and durations in our study, we found comparable results with 3‐year mortality rates of 6% and 73% in non‐metastatic and metastatic breast cancer, respectively.

Dependency, impaired mobility, comorbidities and cognitive impairment are known to have a negative effect on the overall mortality of older patients with cancer, but not on cancer mortality.^9^, ^10^, ^12^, ^13^, ^24^, ^25^, ^26^ This study is therefore the first to highlight an association with cancer death. The first hypothesis is that patients who are frail, present cognitive impairment or have comorbidities are less likely to undergo optimal cancer treatment, thus leading to a higher risk of cancer death. In order to limit side effects and complications in this population, physicians may often prefer to reduce the treatment intensity (i.e. lower dose levels or single‐drug chemotherapy rather than combination).^27^, ^28^, ^29^ In addition, physicians may decide not to start treatment in some patients with cognitive impairment.^30^ Our results are consistent with this hypothesis for middle‐term mortality. Second, cancer per se may influence directly dependency and impaired mobility. A study of women with ovarian cancer suggested that loss of independence was due to diurnal cortisol dysregulation only in patients with advanced disease.^31^ In accordance, reduced mobility was associated with cancer death only in metastatic patients in our study. We found that the risk of cancer death related to comorbidities, alteration of PS and loss of independency decreased with time. Regarding alteration of PS, and loss of independency, this may suggest changes through time (death or recovery). It may suggest a close monitoring of patients with these profiles in the first months of treatment and a standard follow‐up afterwards.

Underweight was not associated with cancer mortality, but more specific nutritional parameters could not be used because data were missing. Obesity was associated with a lower risk of cancer death in our study only in the middle term. Although a causal relationship is subject to debate, the protective effect of overweight and/or obesity in patients with cancer (the so‐called ‘obesity paradox’) has already been described.^32^, ^33^, ^34^

Our sensitivity analyses produced similar results as the main analyses, supporting the robustness of our findings.

The present study has a number of limitations. All patients had been referred to a geriatrician for therapeutic decision, which might have introduced a selection bias. Therefore, we may have overestimated the specific‐cancer mortality rate. Second, misclassification of the cause of death is possible because death certificates are not always filled out correctly. Nevertheless, the degree of agreement between the cause of death recorded on the death certificate and that found in an autopsy is relatively high for cancer (81%).^35^ The ELCAPA‐19 study has some strengths. To our knowledge, our large multicentre cohort study is the first to assess cancer mortality in this population and the impact of geriatric factors on cancer mortality. The use of a competing risk method was appropriate for estimating the crude incidence of death attributable to cancer and the Fine‐Gray regression model is particularly well suited for estimating a patient's clinical prognosis.^36^ Lastly, our results emphasise the importance of geriatric factors in the management of older patients with cancer, notably for the individual estimation of specific oncologic outcomes.

A pragmatic approach might involve the inclusion of geriatric factors in oncologic prognostic scores.^37^, ^38^, ^39^ There is a need to conduct more dedicated trials with older patients with cancer, i.e. taking into account of both unfavourable oncologic and geriatric factors.^40^, ^41^ These trials should also collect data on cancer‐specific deaths. Furthermore, the potential causal role of geriatric factors in cancer deaths warrants further investigation, for example using structural equation modelling.

In conclusion, the majority of older adults with cancer referred for pre‐therapeutic GA die of cancer and not from other causes. An altered general status, loss of independency, and comorbidities were independently associated with cancer death at short and middle term, regardless of metastatic status. Mobility impairment was independently associated with cancer death at short and middle term in metastatic patients. Patients with obesity were less likely to die from cancer than normal‐weight patients at middle term.

## AUTHOR CONTRIBUTIONS

**Déborah Assouan:** Formal analysis (equal); methodology (equal); software (equal); visualization (equal); writing – original draft (equal); writing – review and editing (equal). **Elena Paillaud:** Conceptualization (equal); funding acquisition (equal); investigation (equal); project administration (equal); writing – review and editing (equal). **Philippe Caillet:** Conceptualization (equal); funding acquisition (equal); investigation (equal); project administration (equal); resources (equal); writing – review and editing (equal). **Amaury Broussier:** Investigation (equal); resources (equal); writing – review and editing (equal). **Emmanuelle Kempf:** Investigation (equal); resources (equal); writing – review and editing (equal). **Maxime Frelaut:** Investigation (equal); resources (equal); writing – review and editing (equal). **Etienne Brain:** Funding acquisition (equal); investigation (equal); resources (equal); writing – review and editing (equal). **Emmanuelle Lorisson:** Investigation (equal); resources (equal); writing – review and editing (equal). **Clelia Chambraud:** Data curation (equal); software (equal); writing – review and editing (equal). **Sylvie Bastuji‐Garin:** Funding acquisition (equal); methodology (equal); writing – original draft (equal); writing – review and editing (equal). **Olivier Hanon:** Funding acquisition (equal); investigation (equal); resources (equal); writing – review and editing (equal). **Florence Canouï‐Poitrine:** Conceptualization (equal); funding acquisition (equal); methodology (equal); project administration (equal); resources (equal); supervision (equal); writing – original draft (equal); writing – review and editing (equal). **Marie Laurent:** Conceptualization (equal); investigation (equal); project administration (equal); resources (equal); writing – original draft (equal); writing – review and editing (equal). **Claudia Martinez‐Tapia:** Conceptualization (equal); formal analysis (equal); methodology (equal); software (equal); visualization (equal); writing – original draft (equal); writing – review and editing (equal).

## FUNDING INFORMATION

This work was supported by the French National Cancer Institute (Institut National du Cancer, INCa), Canceropôle Ile‐de‐France and Gerontopôle Ile‐de‐France (Gerond'If). None of the funding bodies had any role in the design and conduct of the study; collection, management, analysis and interpretation of the data; preparation, review and approval of the manuscript; or the decision to submit the manuscript for publication.

## CONFLICT OF INTEREST STATEMENT

The authors declare no conflict of interest.

## ETHICS STATEMENT

The study was conducted in accordance with the Declaration of Helsinki and approved by the local independent ethics committee (CPP Ile‐de‐France I, Paris, France).

## PATIENT CONSENT STATEMENT

All patients provided oral informed consent prior to inclusion.

## CLINICAL TRIAL REGISTRATION

NCT02884375 (ClinicalTrials.gov).

## Supporting information


Data S1:

## Data Availability

Restrictions apply to the availability of these data. Data described in the manuscript will be made available upon request pending approval of the legal sponsor i.e. APHP.
