# Supplementary material for: Cancer mortality and competing causes of death in older adults with cancer: A prospective, multicentre cohort study (ELCAPA‐19)
Source: Cancer Med. 2023 Nov 8;12(22):20940–52. doi: 10.1002/cam4.6639 (PMC10709739; doi:10.1002/cam4.6639)
Supplement: Supplementary file 1 — Data S1: [file CAM4-12-20940-s001.docx]

**Supplementary data**

**
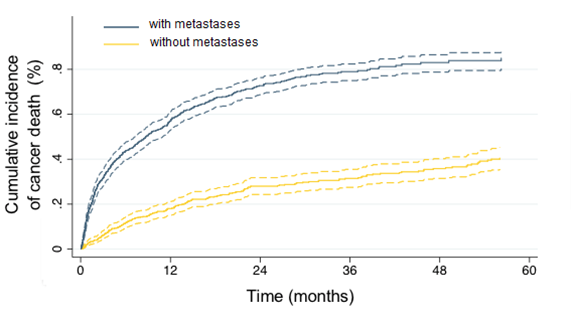
**

**Supplementary Figure 1.** Cumulative incidence (95% CI) of cancer deaths stratified by metastatic status.

**Supplementary Table 1.** Geriatric factors associated with cancer death in patients with a solid tumour and specific treatment for cancer, at 6 months and 3 years, in multivariate analyses (Fine and Gray model) by metastatic status (N=871).

|  | **At 6 monts** | | | | | | |  | **At 3 years** | | | | | | |
| --- | --- | --- | --- | --- | --- | --- | --- | --- | --- | --- | --- | --- | --- | --- | --- |
|  | Non-metastatic | | |  | Metastatic | | |  | Non-metastatic | | |  | Metastatic | | |
| Variable | aSHR | 95%CI | P-value |  | aSHR | 95%CI | P-value |  | aSHR | 95%CI | P-value |  | aSHR | 95%CI | P-value |
| ECOG-PS |  |  |  |  |  |  |  |  |  |  |  |  |  |  |  |
| 0-1 |  |  | 0.70 |  |  |  | 0.006 |  |  |  | 0.79 |  |  |  | <0.001 |
| 2 | 0.80 | 0.31-2.07 | 0.65 |  | 1.40 | 0.79-2.47 | 0.25 |  | 0.84 | 0.48-1.49 | 0.56 |  | 1.16 | 0.78-1.73 | 0.47 |
| 3-4 | 0.50 | 0.09-2.78 | 0.42 |  | 2.88 | 1.50-5.56 | 0.002 |  | 1.09 | 0.43-2.76 | 0.86 |  | 3.28 | 1.93-5.56 | <0.001 |
| Mobility, TGUG |  |  |  |  |  |  |  |  |  |  |  |  |  |  |  |
| ≤ 20 s |  |  | 0.14 |  |  |  | 0.017 |  |  |  | 0.17 |  |  |  | 0.033 |
| > 20 s | 2.05 | 0.74-5.71 | 0.17 |  | 1.69 | 1.00-20.9 | 0.049 |  | 1.73 | 0.96-3.12 | 0.068 |  | 1.48 | 1.03-2.14 | 0.035 |
| Unable to perform the test | 6.81 | 0.97-47.5 | 0.053 |  | 2.84 | 1.37-5.89 | 0.005 |  | 1.38 | 0.33-5.83 | 0.66 |  | 2.04 | 1.12-3.70 | 0.019 |
| Dependency for ADLs (≤5/6) b | 1.71 | 0.79-3.70 | 0.18 |  | 2.97 | 1.71-5.17 | <0.001 |  | 1.76 | 1.03-3.01 | 0.039 |  | 2.74 | 1.66-4.51 | <0.001 |
| CIRS-G ≥ 13 b | 2.69 | 1.16-6.24 | 0.021 |  | 1.77 | 1.11-2.83 | 0.017 |  | 1.35 | 0.85-2.15 | 0.21 |  | 2.31 | 1.36-3.93 | 0.002 |
| Cognitive impairment c | 1.20 | 0.43-3.33 | 0.72 |  | 1.18 | 0.70-1.96 | 0.54 |  | 1.20 | 0.70-2.05 | 0.51 |  | 1.05 | 0.70-1.57 | 0.81 |
| Abnormal mini-GDS (≥1/4) | 1.47 | 0.60-3.60 | 0.39 |  | 1.03 | 0.64-1.65 | 0.91 |  | 1.16 | 0.69-1.95 | 0.58 |  | 0.97 | 0.71-1.32 | 0.85 |
| BMI (kg/m2) |  |  |  |  |  |  |  |  |  |  |  |  |  |  |  |
| < 21 |  |  | 0.91 |  |  |  | 0.029 |  |  |  | 0.34 |  |  |  | 0.083 |
| [21-25[ | 0.72 | 0.11-4.88 | 0.74 |  | 1.65 | 1.00-2.70 | 0.048 |  | 0.85 | 0.40-1.78 | 0.67 |  | 1.15 | 0.80-1.67 | 0.46 |
| [25-30[ | 0.96 | 0.36-2.55 | 0.93 |  | 0.80 | 0.48-1.34 | 0.40 |  | 0.62 | 0.37-1.06 | 0.083 |  | 0.71 | 0.51-0.99 | 0.044 |
| ≥ 30 | 1.31 | 0.43-3.98 | 0.64 |  | 0.74 | 0.33-1.64 | 0.46 |  | 0.65 | 0.32-1.32 | 0.23 |  | 0.87 | 0.56-1.36 | 0.54 |

Abbreviations: aCSHR: adjusted Cox specific hazard model; CI: confidence interval; ECOG-PS: Eastern Cooperative Group performance status; ADL: activities of daily living; TGUG: Timed-Get-Up-and-Go score; GDS: geriatric depressions; BMI: body mass index

Note: Adjusted subhazard ratios of cognitive impairment, mini-GDS and BMI correspond to those from model 1.
